# A new hepatitis E virus genotype 2 strain identified from an outbreak in Nigeria, 2017

**DOI:** 10.1186/s12985-018-1082-8

**Published:** 2018-10-23

**Authors:** Bo Wang, Olusola Anuoluwapo Akanbi, Dominik Harms, Olufisayo Adesina, Folakemi Abiodun Osundare, Dhamari Naidoo, Isabel Deveaux, Opeayo Ogundiran, Uzoma Ugochukwu, Nwando Mba, Chikwe Ihekweazu, C.-Thomas Bock

**Affiliations:** 1 Division of Viral Gastroenteritis and Hepatitis Pathogens and Enteroviruses, Department of Infectious Diseases, Robert Koch Institute, 13353 Berlin, Germany; 2 Ladoke Akintola University of Technology, Ogbomoso, Oyo State P.M.B 4000 Nigeria; 3 Infectious Hazard Management Department, World Health Organization, Geneva, Switzerland; 4 Nigeria Centre for Disease Control, Plot 801, Ebitu Ukiwe Street, Jabi, Abuja, Nigeria; 5 Institute of Tropical Medicine, University of Tübingen, 72074 Tübingen, Germany

**Keywords:** Hepatitis E virus, HEV genotype 2, HEV subtype 2b, Outbreak, Nigeria, Complete genome

## Abstract

**Background:**

In 2017 the Nigerian Ministry of Health notified the World Health Organization (WHO) of an outbreak of hepatitis E located in the north-east region of the country with 146 cases with 2 deaths. The analysis of the hepatitis E virus (HEV) genotypes responsible for the outbreak revealed the predominance of HEV genotypes 1 (HEV-1) and 2 (HEV-2). Molecular data of HEV-2 genomes are limited; therefore we characterized a HEV-2 strain of the outbreak in more detail.

**Finding:**

The full-length genome sequence of an HEV-2 strain (NG/17–0500) from the outbreak was amplified using newly designed consensus primers. Comparison with other HEV complete genome sequences, including the only HEV-2 strain (Mex-14) with available complete genome sequences and the availability of data of partial HEV-2 sequences from Sub-Saharan Africa, suggests that NG/17–0500 belongs to HEV subtype 2b (HEV-2b).

**Conclusions:**

We identified a novel HEV-2b strain from Sub-Saharan Africa, which is the second complete HEV-2 sequence to date, whose natural history and epidemiology merit further investigation.

## Main text

Hepatitis E virus (HEV) is the prototype of the family *Hepeviridae* and a common causative agent of acute viral hepatitis. HEV is a small, (non)enveloped spherical particle of about 34 nm in diameter harboring a single stranded, positive sense RNA genome of approximately 7.5 kb [1]. Eight HEV genotypes are recognized within the species *Orthohepevirus* A based on the pairwise distances of entire viral genomes (HEV-1 to HEV-8). The different HEV genotypes have various reservoirs, distinct distribution, and transmission patterns. Four major HEV genotypes (HEV-1 to HEV-4) are well recognized as human pathogens while HEV-5 and HEV-6 have been detected only in wild boars so far. HEV-7 from dromedary camels has been reported to infect humans and cause chronic hepatitis E. HEV-8 is identified in Bactrian camels with an unknown zoonotic potential [2–4]. HEV-1 and HEV-2 are transmitted through the waterborne/fecal-oral route and responsible for large HEV outbreaks and epidemics in endemic areas like the Indian subcontinent and Africa, whereas HEV-3 and HEV-4 are linked to zoonotic transmission causing sporadic infections mainly in industrialized countries [5]. HEV-2 was firstly identified during a hepatitis E outbreak in Mexico in 1986 while the complete genome sequence (HPENSSP, GenBank accession No. M74506) was subsequently characterized [6]. Recently, additional HEV-2 full-length genome sequences were obtained from an individual patient of same Hepatitis E outbreak in Mexico (Mex-14, KX578717) showing 99.5% nucleotide identity to M74506 [7]. However, the sequences for M74506 and KX578717 are from the same isolate. Since M74506 has been referred to as Mexico [8], Mexico-14 [9], and Telixtac-14 [10], the stool sample was re-analysed at the Paul Ehrlich Institute (PEI), Germany, with the isolate designation Mex-14, and the sequences were submitted to GenBank receiving the accession number KX578717. Additionally, several studies have reported that HEV-2 is distributed in Africa. However, only partial ORF2 gene sequences were amplified [11–14]. According to the report of Lu et al. and the International Committee on Taxonomy of Viruses (ICTV) *Hepeviridae* Study Group, Mex-14 is the proposed HEV subtype 2a (HEV-2a) reference sequence, and HEV subtype 2b (HEV-2b) was assigned to the partial sequences AF173231 and AF173232 from Nigeria and AY903950 from Chad [15, 16].

In June 2017, the Nigerian Ministry of Health reported to the World Health Organization (WHO) an HEV outbreak in north-east Nigeria, with 146 laboratory confirmed cases and two outbreak-associated cases of death in pregnant [17]. In order to identify the corresponding viral pathogens of this hepatitis E outbreak, we determined HEV genotypes from outbreak samples. The genotyping results showed mainly HEV-1 and HEV-2 strains being predominant within the outbreak, the genotype distribution of isolates from this outbreak as determined by the Nigerian center of disease control (NCDC) and Robert Koch Institute (RKI) was 40% HEV-1 and 60% HEV-2. However, a number of HEV-positive outbreak samples could not genotyped. Since full-length genome sequences of HEV-2 strains are rare, we here report the full-length genome sequence of the HEV-2 strain (NG/17–0500) from an isolate of the Nigerian outbreak. The virus was detected from an individual from Borno state, Nigeria, and initially tested serologically positive for HEV using Wantai HEV-IgM Rapid Test and Wantai HEV-IgM ELISA (Sanbio, Uden, Netherland).

Viral RNA from an anonymized serum sample (NG/17–0500) was extracted using High Pure Viral Nucleic Acid Kit (Roche, Mannheim, Germany) following cDNA synthesis using SuperScript™ III First-Strand Synthesis System (Invitrogen, Carlsbad, CA, USA) according to the manufacturer’s instructions. Molecular approaches with sensitive real-time PCR and consensus nested PCR assays were conducted as described recently [18]. To amplify the entire genome sequence of NG/17–0500 and to verify the geno/subtyping results, universal primers were designed based on 38 complete HEV-1 and HEV-2 sequences from the GenBank database. Using genome walking method, gene-specific primers were designed to amplify the gaps. Primers and probe used are listed in Table 1. The complete viral genome of NG/17–0500 was amplified using KAPA HiFi HotStart ReadyMix PCR kit (Roche, Mannheim, Germany). 5′ and 3′ sequences were determined using 5′ and 3′ rapid amplification of cDNA ends (Roche, Mannheim, Germany). Sense and antisense strands of PCR amplicons were sequenced with BigDye Terminator version 3.1 cycle sequencing kit (Thermo Fisher Scientific, USA). Whole genome sequence was assembled and analyzed using Geneious software version 10.0.5. (Biomatters Limited, Auckland, New Zealand) [19].

**Table 1:** Primers used for HEV quantification, genotyping, and complete genome sequencing

Primer^a^	Sequence (5′-3′)	Location^b^	Use	Reference
HEV-07_f	GGTGGTTTCTGGGGTGAC	5261–5278	HEV-1 to HEV-4 quantification	[18]
HEV-TM3_f	FAM-TGATTCTCAGCCCTTCGC-MGB	5284–5301		
HEV-08_r	AGGGGTTGGTTGGATGAA	5330–5313		
HEV-38_f	GARGCYATGGTBGAGAARG	4084–4102	HEV-1 to HEV-4 genotyping in ORF1	[18]
HEV-39_r	GCCATRTTCCARACRGTRTTCC	4622–4601		
HEV-37_f	GGTTYCGYGCYATTGARAARG	4277–4297		
HEV-27_r	TCRCCRGARTGYTTCTTCC	4583–4565		
HEV-30_f	CCGACAGAATTRATTTCGTCGG	6296–6317	HEV-1 to HEV-4 genotyping in ORF2	
HEV-32_f	GTCTCRGCCAATGGCGAGCCRRC	6350–6372		
HEV-31_r	GTYTTRGARTACTGCTGR	6750–6733		
HEV-266_f	GCARGCTGCTCTRGCWGCGGC	78–98	HEV-2 complete genome sequencing	This study
HEV-274_f	TGGTGGTTAGGCCTTTTCTCTC	122–143		
HEV-275_f	CCGATCCAGCGTGTCATACATA	223–244		
HEV-267_r	GGRGCWGWRTACCARCGCTG	392–373		
HEV-268_f	AYCTYCGYGGYATTAGCTAYAAGG	1055–1078		
HEV-276_r	CGTTGATGGCAAATTGTGAGGT	1178–1157		
HEV-277_f	ATCTCTCGTCTCTACAGCTGGT	1246–1267		
HEV-278_f	GGGCCGTCAGTTGCAATTTTAT	1299–1320		
HEV-283_f	GTAGCTGCCGGACTATTGCT	1397–1416		
HEV-270_r	ARCCACYKCATAAARCARC	1457–1439		
HEV-284_f	ACCAGGGCCATGACAATGAG	1508–1527		
HEV-285_r	GAGGCCTGGTCAGCAACTAG	2186–2164		
HEV-271_f	AACCCMAAGAGGCUYGAGGC	2620–2639		
HEV-272_f	GCCTGGGARCGKAAYCAYCG	2734–2753		
HEV-279_f	TGTTCAACGTAGGATGATCCGG	2833–2812		
HEV-280_f	TTTGAGCATACTGGTCTGGTCC	3220–3241		
HEV-273_r	CARCGRUGKGURACAUGCCACC	3296–3275		
HEV-235_r	CYGCCTGGGTGAACACTAG	3421–3403		
HEV-265_f	ATGGGGACGCCTATGATGAATC	4337–4358		
HEV-282_r	TTCTGGGTCGAGTCAAACTCAG	4439–4418		
HEV-281_r	CACTCCTCCATAATAGCGCACT	4481–4460		
HEV-286_f	TTCTGCTGTTGCTCCTCCTG	5169–5188		
HEV-301_f	AGACGTCTGGTGTTGCTGAG	5937–5956		
HEV-288_r	TTTACTGTCGGCTCGGCATT	6384–6365		
HEV-287_r	GCTGGGCATTCTCCACAGAT	6413–6394		
HEV-233_f	GCCTSTTTTGTGATGCGCG	6755–6773		

^a^Forward primer designations end with _f; reverse primer designations end with _r

^b^Numbering is according the HEV prototype strain Burma (GenBank accession No. M73218)

**Fig. 1:**
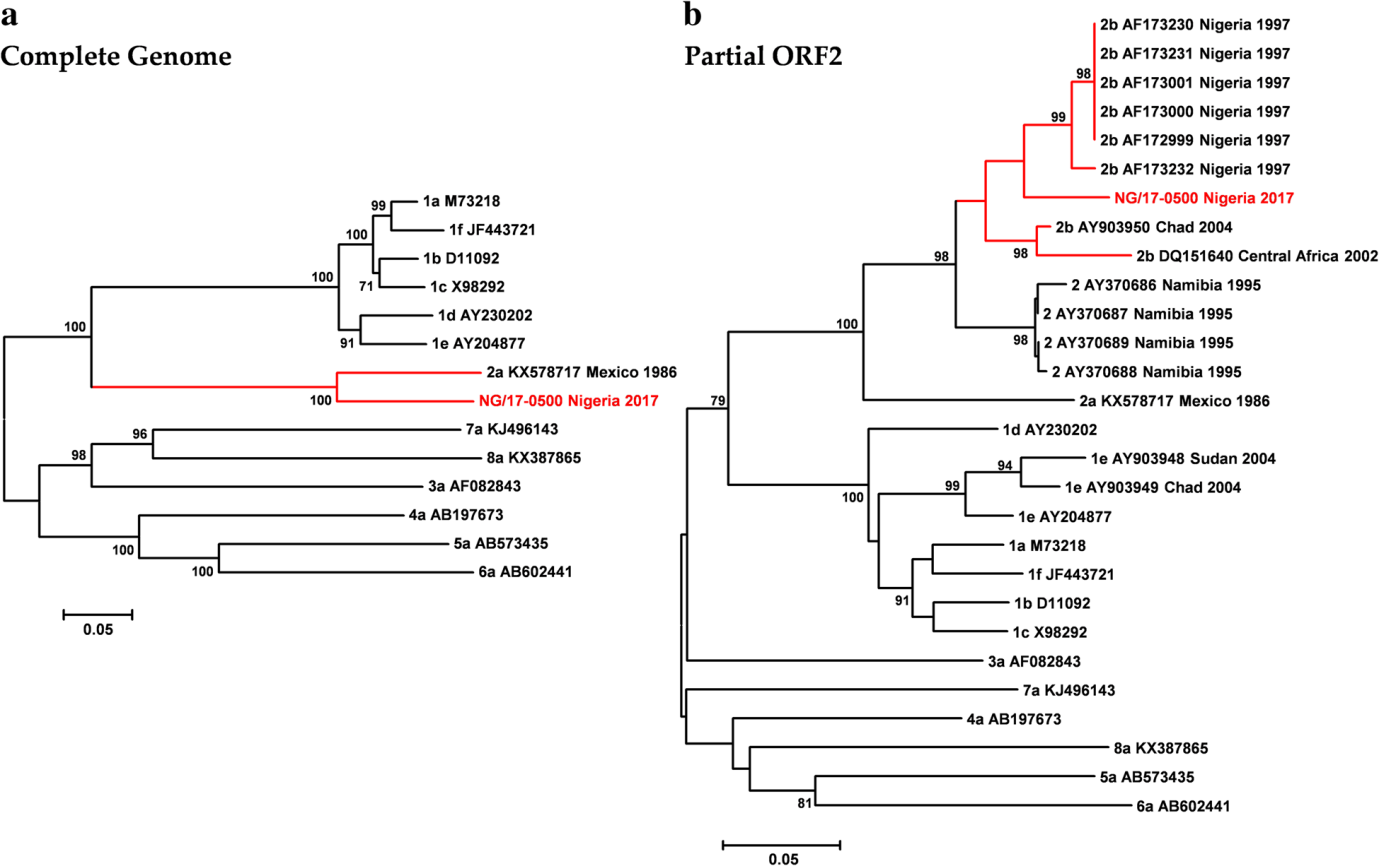
Phylogenetic relationships of NG/17–0500 within the species *Orthohepevirus* A. HEV-2 strains are designated with geno/subtype, accession number, country, and collection year. NG/17–0500 of this study is shown in red. Phylogenetic analyses were performed with MEGA software version 7.0.26. Maximum likelihood trees based on General Time Reversible model with Gamma distributed with Invariant sites was inferred. The values at nodes indicate the bootstrap values (using 1000 replications). Values below 70% are hidden for clarity of presentation. Reference sequences for HEV genotypes were as proposed from the ICTV *Hepeviridae* Study Group. Nucleotide (nt) and amino acid (aa) sequences were aligned using MAFFT software version 7.222. **a** Phylogenetic relationships based on complete genome sequences of representative HEV reference strains. HEV-2 strains are highlighted with red branches. **b** Phylogenetic relationships based on 641 nt of ORF 2 corresponding to nt positions 6453 to 7093 (numbered according to the HEV prototype strain from Burma GenBank accession No. M73218). HEV-2b strains are highlighted with red branches

Real-time PCR assay targeting the HEV ORF2 and ORF3 overlapping region (ORF2/3) demonstrated viremic HEV infection with a viral load of 1.2 × 10E + 7 IU/mL. Sequence analysis of partial ORF1 and ORF2 genes indicated that NG/17–0500 preliminary belongs to HEV-2. After de novo assembly of the amplicons, the NG/17–0500 full-length sequence showed 7198 nucleotides, excluding the 3′-poly (A) tail, with a G + C content of 57.5% harboring the typical 3 HEV ORFs. Phylogenetic analysis of the complete genome sequence revealed that NG/17–0500 grouped with HEV-2 Mex-14 strain (Fig. 1a), and this was all true for phylogenetic analysis of individual ORFs (data not shown). These relationships were also observed for sequence identities between NG/17–500 and other HEV complete genome or nucleotide or amino acid sequence identities of individual ORFs (Table 2).

**Table 2:** Nucleotide and amino acid sequence identities between NG/17–0500 and reference HEV strains within the family *Hepeviridae*^a^

*Hepeviridae*	Degree of identity (%)						
	Complete genome	ORF1		ORF2		ORF3	
	nt^b^	nt	aa	nt	aa	nt	aa
HEV-1	75.0	72.3	83.5	81.0	94.8	90.9	87.8
HEV-2	83.5	82.3	92.7	86.8	98.2	94.1	91.1
HEV-3	73.1	71.2	81.5	78.7	91.2	83.9	76.4
HEV-4	73.5	71.1	80.6	80.7	91.1	80.6	78.9
HEV-5	72.6	70.1	80.1	78.6	89.1	75.0	74.1
HEV-6	72.8	70.9	79.4	78.0	88.5	75.5	70.5
HEV-7	72.3	69.8	80.6	78.8	89.8	79.0	79.8
HEV-8	72.2	70.0	79.9	77.9	89.4	78.5	81.6
Avian HEV	46.3	44.9	41.3	46.2	43.3	25.6	21.1
Rat HEV	52.1	50.1	48.0	55.1	54.8	38.1	27.1
Bat HEV	47.2	45.8	41.6	55.1	48.1	30.6	15.9

^a^The sequences were aligned using MAFFT software version 7.222. The evolutionary analyses were conducted using MEGA 7 software version 7.0.26. The GenBank accession numbers are for HEV-1 (M73218), HEV-2 (KX578717), HEV-3 (AF082843), HEV-4 (AJ272108), HEV-5 (AB573435), HEV-6 (AB602441), HEV-7 (KJ496143), HEV-8 (KX387865), Avian HEV (AY535004), Rat HEV (GU345042), and Bat HEV (JQ001749)

^b^nt and aa represent nucleotide and amino acid, respectively

**Fig. 2:**
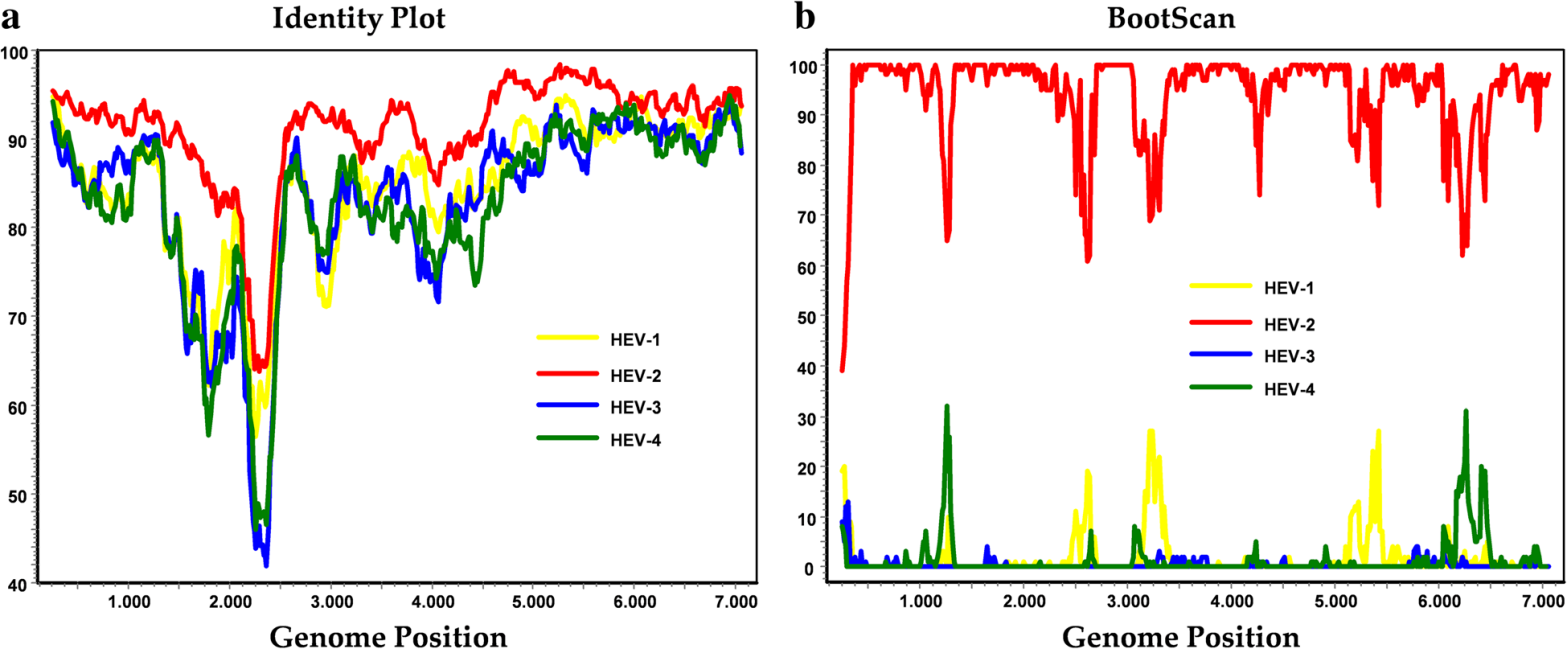
Detection of potential recombination events of NG/17–0500 within HEV-1 to HEV-4. **a** Identity Plot and **b** BootScan analyses of full-length sequences were performed using SimPlot software program version 3.5.1 with an F84 distance model, a sliding window size of 300 base pairs and a step size of 15 base pairs increment. Positions containing gaps were stripped from the alignment

Due to inadequate sanitation and lack of clean drinking water, hepatitis E is a severe public health issue in several regions of Africa [20]. Partial HEV-2 sequences have been reported from Sub-Sahara African countries mainly during HEV outbreaks including Namibia in 1995 [12], Sudan in 2004 [13], and the Central African Republic in 2002 [14]. In addition, a single study reported of the analysis of HEV isolates from ten sporadic cases in Port-Harcourt city, southern Nigeria in 1997. Phylogenetic analysis of partial ORF2 fragments indicated that the Nigerian isolates from 1997 are most closely related to the HEV-2a reference Mex-14 strain and have been proposed as HEV-2b [11, 15, 16]. In this regard, comparison of NG/17–0500 sequence to the previously characterized Nigerian HEV-2b isolates displayed a 91.2% to 92.2% nt identity. Phylogenetic analysis of the availability of data of partial HEV-2 sequences from Sub-Saharan Africa showed that NG/17–0500 clustered with proposed HEV-2b sequences, indicating NG/17–0500 belongs to HEV-2b (Fig. 1b). Comparison of partial ORF2 sequences of NG/17–0500 to Chad (AY903950) and Central African Republic (DQ151640) HEV-2b isolates shared 90.3% and 88.4% identity, respectively. No evidence of recombination in NG/17–500 was detected by either Identity Plot or Bootscan analysis (Fig. 2). The complete genome sequence of NG/17–0500 has been deposited in GenBank under the accession number MH809516.

In conclusion, to the best of our knowledge the novel HEV-2b strain NG/17–0500 from Nigerian hepatitis E outbreak 2017 represents the first complete HEV-2 genomic sequence from Sub-Sahara Africa and the second complete HEV-2 sequence worldwide, which contributes to our knowledge of the diversity of HEV-2. Nevertheless, the natural history of NG/17–0500 requires further comprehensive genetic and epidemiological analyses.

## Abbreviations

aa: Amino acid
HEV: Hepatitis E virus
HEV-1 to HEV-8: Hepatitis E virus genotype 1 to hepatitis E virus genotype 8
HEV-2a and HEV-2b: Hepatitis E virus subtype 2a and 2b
ICTV: International Committee on Taxonomy of Viruses
NCDC: Nigerian center of disease control
nt: Nucleotide
ORF: Open reading frame
PEI: Paul Ehrlich Institute
RKI: Robert Koch Institute
WHO: World Health Organization
